# Editorial: Advances in the treatment of Hodgkin lymphoma

**DOI:** 10.3389/fonc.2023.1164081

**Published:** 2023-02-28

**Authors:** Matthew Mei, Guilherme Perini, Radhakrishnan Ramchandren

**Affiliations:** ^1^ Department of Hematology and Hematopoietic Cell Transplantation, City of Hope, Duarte, CA, United States; ^2^ Hospital Israelita Albert Einstein, São Paulo, Brazil; ^3^ Division of Hematology/Oncology, University of Tennessee Health Science Center, Knoxville, TN, United States

**Keywords:** Hodgkin lymphoma, stem cell transplantation, brentuximab vedotin, chemotherapy, elderly

Recent years have seen numerous advances in classical Hodgkin lymphoma (cHL) both from the standpoint of understanding its unique disease biology and in clinical practice, with an increasing number of patients treated effectively with novel combinations across multiple lines of therapy. For instance, incorporation of brentuximab vedotin (BV) in the frontline setting has now been shown to extend overall survival as compared to ABVD (1), and second-line regimens incorporating programmed cell death-1 (PD-1) blockade have delivered unprecedented response rates (2–4). However, in spite of the fact that the large majority of patients with newly diagnosed cHL are ultimately cured, the notion that cHL has been solved is false, and there remains significant room for further innovation.

This Frontiers special section on Advances in the Treatment of Hodgkin Lymphoma includes five manuscripts that highlight some of the progress made as unmet needs remain in cHL. Song et al. report on the outcomes of penpulimab, a novel anti-PD-1 monoclonal antibody, in patients with relapsed/refractory (r/r) cHL. In distinction to other approved anti-PD-1 therapies in cHL (nivolumab, pembrolizumab in the U.S.; tislelizumab,sintilimab, and camrelizumab in China) which are IgG4 monoclonal antibodies, penpulimab uses an IgG1 construct. Moreover, it is further engineered with an Fc mutation that eliminates the Fc receptor, which prevents antibody-dependent cell-mediated cytotoxicity. The overall response rate (89%) in pre-treated patients who had at least two prior lines of therapy compares favorably to other anti-PD-1 agents; notably, no patient had prior BV, however. Toxicity (grade 3+ irAE of 4.3%) also seemed low compared to other checkpoint inhibitors in use, and a randomized phase 3 trial of penpulimab vs. investigator’s choice therapy is underway in China.

Li et al. report on a single fascinating case of a child with a germline titin (TTN) mutation and severely depressed ejection fraction at the time of being diagnosed with advanced stage cHL. As pathogeneic TTN mutations appear to be highly correlated with anthracycline-induced cardiotoxicity, he was treated with an anthracycline-free regimen including BV and subsequently achieved CR, which had been sustained for 11 months at the time of the writing of the manuscript. Delivery of curative therapy without anthracycline use remains elusive in cHL; yet, with a growing appreciation of the long-term cardiotoxic risk of anthracycline exposure (5), this remains an unmet need and an important goal.

Barrett and Collins provide an excellent summary on challenges relating to the treatment of older persons with cHL and the difficult decision-making regarding the optimal choice of therapy in this population as delivery of curative therapy is fraught with baseline comorbidities, yet undertreating the disease in an effort to avoid toxicity also results in poor outcomes. Minimizing the use of bleomycin, the judicious use of intensive salvage therapies such as autologous stem cell transplantation (autoHCT) in eligible patients, and the unmet need for improved therapies for truly unfit patients are all highlighted.

Maranzano and Mead review data and strategies surrounding stem cell transplantation in cHL. They discuss novel salvage therapies as incorporation of BV and/or anti-PD-1 agents has resulted in an unprecedented number of patients proceeding to potentially curative autoHCT in complete remission as well as the role of maintenance therapies after autoHCT. Allogeneic stem cell transplantation (alloHCT) still plays a role in patients who relapse after prior autoHCT but is associated with significant toxicity, particularly when performed shortly after recent anti-PD-1 exposure.

Finally, Ullah et al. comprehensively review the treatment landscape of cHL with a focus on novel therapies and approaches. In addition to reviewing existing approved therapies, they also discuss newer data on epigenetic treatments in cHL, the potentially emerging role of alternative immune checkpoint inhibitors directed against LAG-3 and TIGIT, and cellular therapies including chimeric antigen receptor T-cell (CAR T-cells) and natural killer (NK) cell therapy, both of which have demonstrated clinical efficacy in early phase trials.

## Author contributions

MM conceptualized and wrote the first draft of the manuscript. All authors edited the manuscript and approved the final version.

## References

[1] Ansell SM Radford J Connors JM Długosz-Danecka M Kim WS Gallamini A . Overall survival with brentuximab vedotin in stage III or IV hodgkin’s lymphoma. New Engl J Med (2022) 387(4):310–20. doi: 10.1056/NEJMoa2206125 35830649

[2] Moskowitz AJ Shah G Schöder H Ganesan N Drill E Hancock H . Phase II trial of pembrolizumab plus gemcitabine, vinorelbine, and liposomal doxorubicin as second-line therapy for relapsed or refractory classical Hodgkin lymphoma. J Clin Oncol (2021) 39(28):3109–17. doi: 10.1200/JCO.21.01056 PMC9851707 34170745

[3] Mei MG Lee HJ Palmer JM Chen R Tsai NC Chen L . Response-adapted anti-PD-1-based salvage therapy for Hodgkin lymphoma with nivolumab alone or in combination with ICE. Blood (2022) 139(25):3605–16. doi: 10.1182/blood.2022015423 PMC9227101 35316328

[4] Ding K Liu H Ma J Yang H Cao L Wang H . Tislelizumab with gemcitabine and oxaliplatin in patients with relapsed or refractory classic Hodgkin lymphoma: A multicenter phase II trial. Haematologica (2023). doi: 10.3324/haematol.2022.282266 PMC10388287 36700408

[5] Larsen CM Garcia Arango M Dasari H Arciniegas Calle M Adjei E Rico Mesa J . Association of anthracycline with heart failure in patients treated for breast cancer or lymphoma, 1985-2010. JAMA Netw Open (2023) 6(2):e2254669–e. doi: 10.1001/jamanetworkopen.2022.54669 PMC9898820 36735254

